# Non-collagenous extracellular matrix protein dermatopontin may play a role as another component of transforming growth factor-β signaling pathway in colon carcinogenesis

**DOI:** 10.22038/IJBMS.2021.46422.10720

**Published:** 2021-04

**Authors:** Ariane Sadr-Nabavi, Samaneh Bouromand-Noughabi, Naser Tayebi-Meybodi, Kimia Dadkhah, Nafiseh Amini, Alfons Meindl, Mohammad Reza Abbaszadegan

**Affiliations:** 1 Medical Genetic Research Center (MGRC), School of Medicine, Mashhad University of Medical Sciences, Mashhad, Iran; 2 Department of Human Genetics, School of Medicine, Mashhad University of Medical Sciences, Mashhad, Iran; 3 Iranian Academic Centers for Education, Culture, and Research (ACECR); 4 Department of Pathology, Faculty of Medicine, Mashhad, University of Medical Sciences, Mashhad, Iran; 5 Cancer Molecular Pathology Research Center, Mashhad University of Medical Sciences, Mashhad, Iran; 6 Division of Human Genetics, Immunology Research Center, Avicenna Research Institute, Mashhad University of Medical Sciences, Mashhad, Iran; 7 Frauenklinik der Technischen Universität München, Klinikum rechts der Isar, München, Germany

**Keywords:** Colorectal cancer, Dermatopontin, Real-Time PCR, Sanger sequencing, TGF-β

## Abstract

**Objective(s):**

Dermatopontin (DPT) is an extracellular matrix protein that plays roles in increasing the activity of transforming growth factor-β (TGF-β) and induction of cell quiescence. These roles suggest a tumor suppressor function for DPT. This study aimed to investigate changes in DPT gene expression in colorectal cancer providing a better understanding of its carcinogenesis.

**Materials and Methods:**

We used Matched Tumor/Normal Expression Array and Cancer Profiling Arrays I containing 34 and 7 cases of colorectal cancer and their matched controls, respectively, to test DPT expression. In addition, 38 newly diagnosed cases of colorectal cancer were enrolled and their fresh colonic tumoral and normal specimens were obtained. DPT mRNA expression was analyzed using real-time PCR. In cases with DPT under expression, exonic regions of the DPT gene were sequenced using the Sanger method.

**Results:**

In array samples, DPT expression was decreased in 82.9% (34/41), increased in 12.2% (5/41), and had no changes in 4.9% (2/41). DPT was decreased in 14 fresh samples (36.8%), while 12 cases (31.6%) showed overexpression and the others had no changes. DPT expression showed no significant difference among various tumor grades and stages. The frequencies of DPT overexpression were higher in tumors having lymph node involvement (47.7% vs 28%, *P*=0.59). In 2 cases mutations were detected that may be responsible for decreased expression of DPT.

**Conclusion:**

The similarities between changing patterns of DPT and TGF-β expression in colorectal cancer demonstrate that DPT may act as a pre-receptor component of the TGF-β signaling pathway in colon carcinogenesis.

## Introduction

Colon cancer is the third most common malignancy and the second leading cause of cancer-related mortality worldwide (1). In Iran, it is the third most common cancer in women after skin and breast cancers, and the fifth most common cancer in men (after skin, stomach, bladder, and prostate cancers) (2). The five-year survival rate of colorectal cancer (CRC) is about 90% when diagnosed at an early stage. However, less than 40% of CRC cases are diagnosed in the early stages (3). Currently, the best treatment is surgery, and chemotherapy shows limited success in the treatment of colon cancer (4-6). Various biomarkers are proposed in order to help early diagnosis of CRC (4, 5). 

Dermatopontin (DPT) is a non-collagenous extracellular matrix protein consisting of 183 amino acids with a molecular weight of 22 kDa (7, 8). DPT has a large number of tyrosine residues, which can be sulfated and plays an important role in interaction with other proteins (7). It has been identified that DPT plays roles in cell adhesion, fibrillogenesis of collagen fibers, and also as a downstream target of the vitamin D receptor (7, 9, 10). Besides, DPT increases the biological activity of transforming growth factor-β (TGF-β), which in turn acts as a tumor suppressor in many cancers (7, 11-13). Reduced expression of DPT has been found in various neoplasms, including oral cancer, hepatocellular carcinoma, breast cancer, ovarian cancer, and leiomyoma. Furthermore, its expression has been correlated with lymph node metastasis (14-19).

TGF-βs are cytokines with a unique central role in homeostasis, wound healing, fibrosis, angiogenesis, carcinogenesis, and cell differentiation. All of these activities are coordinated through the same signaling pathway. In this pathway, after phosphorylation of Smad2 and Smad3, a heteromeric complex with Smad4 is generated which binds to specific regulatory elements on target genes. It is now believed that TGF-β inhibits the early stages of tumorigenesis, while in the later stages it is involved in the progression of cancer (20). Disruption in TGF-β or the components of its signaling pathway is a fundamental part of the pathogenesis of several molecular types of CRC. In addition, there are clinical pieces of evidence addressing its strong effect on the prognosis of patients after surgical therapy (12). 

To the best of our knowledge, changes in DPT expression have not been so far studied in colon cancer. Given the high prevalence of TGF-β signaling pathway alterations in colon cancers and DPT function as a possible enhancer of this pathway; we decided to evaluate DPT changes in CRC patients. It is hoped that by studying the changes in DPT, its role in the development of colon cancer, metastasis and its correlation with prognosis, based on grading and staging, is elucidated, thereby providing a better understanding of the molecular pathogenesis of CRC. 

## Materials and Methods

Matched Tumor/Normal expression Array and Cancer Profiling Array I were used for the expression study. The Matched Tumor/Normal expression Array consists of cDNA pairs from 10 different tissues and the Cancer Profiling Array I contains multiple cDNA pairs from 13 different tissues which were synthesized from human tumoral and corresponding normal tissues. Each pair was independently normalized based on the expression of three housekeeping genes and immobilized in separate dots. Detailed clinical information and a complete list of tissues can be found on the provider’s website (WWW.clontech.com/techinfo/manuals/pdf/7841-1.pdf). Hybridizations using 25 ng of a gene-specific 32P-labelled cDNA probe digested from Unigenec DNA clones were performed according to the manufacturer’s recommendations. 

The tumor/normal intensity ratio was calculated using a STORM-860 phosphoimager (Molecular Dynamics, Eugene, OR, USA) and normalized against the background. We defined a candidate gene as differentially expressed in a given tumor entity if common deregulation (two-fold up- or down-regulation) was detectable in at least 20% of analyzed tumor tissue samples.

***Sample collection for real -time PCR and sequencing***

Our studied population included 38 histologically confirmed colorectal adenocarcinoma patients, who had undergone colectomy before chemo/radio-therapy at Omid, Imam Reza, and Ghaem University Hospitals (Mashhad University of Medical Sciences, Mashhad, Iran). The study protocols were approved by the Research Ethics Committee in Mashhad University of Medical Sciences, Mashhad, Iran.

Fresh tumoral and normal specimens were obtained within 1 hr after surgery by the pathologists and kept in RNAlater solution (Qiagen, Hilden, Germany) at -20 °C until extraction. The sites of tumoral and normal tissue extraction were examined microscopically in order to make sure that at least 70% of tumoral cells and no or rare tumor infiltration are present, respectively (macro-dissection). 

***RNA extraction and cDNA synthesis***

Total RNA from tumoral and normal samples was extracted using the RNeasy Mini kit (74104, Qiagen, Germany) according to the manufacturer’s instructions. The integrity and purity, as well as quantity of the extracted RNA, were evaluated by an ultraviolet spectrophotometer and agarose gel electrophoresis.

Total RNA was reversely transcribed using the Revert-Aid first-strand cDNA synthesis kit and oligo(dT)18 primers (K1631, Fermentas, Vilnius, Lithuania). In brief, 0.5 µg of total RNA plus 0.5 µg of oligo(dT)18 and 9.5 µl of DEPC-treated water in a total volume of 12.5 µl were mixed. After removing secondary RNA structures by heating at 65 °C for 5 min, the mixture was placed on ice until the addition of the cDNA synthesis mix (4 µl 5X cDNA synthesis buffer, 0.5 (0.375) mM dNTP mix, 20 U Ribolock ribonuclease inhibitor, and 200 U reverse transcriptase). The mixture contained Ribolock ribonuclease inhibitor which inhibits the activity of RNase A, B, and C. 

The final mix (20 µl) was incubated for 60 min at 42 °C and finally 10 min at 70 °C for enzyme inactivation.

***Real-time polymerase chain reaction***

A set of mRNA-specific oligonucleotide primers for dermatopontin (gene symbol: DPT, accession number: NM_001937) (gene of interest) and a set of gene-specific oligonucleotide primers for the housekeeping glyceraldehyde-3-phosphate dehydrogenase gene (GAPDH) were designed (Table 1). Primer-BLAST was performed, confirming the specificity of the primers (www.blast.ncbi.nlm.nih.gov). Real-time polymerase chain reactions were performed by the Stratagene Mx-3000P real-time thermocycler using the software version 3.00 (Stratagene, La Jolla, CA, USA) and SYBR green methods via SYBR Green Master Mix (K0221, Fermentas, Lithuania). The reaction mix was prepared by adding 10 µl SYBR Green Master Mix (2X), 0.25 µl of each primer (10 pmol/µl), 2 µl cDNA, and 7.5 µl of nuclease-free water in a total volume of 20 µl. The tests were done in duplicate. Two negative controls (without cDNA), one of which contained GAPDH primers and the other DPT primers, were included in each PCR assay.

The thermal cycle conditions of the study were as follows:

Uracil-DNA Glycosidase pre-treatment: 3 min at 50 °C 

(to remove all dU-containing amplicons carried over from previous reactions)

Initial denaturation: 10 min at 95 °C

40 cycles: 

- Denaturation: 95 °C for 30 sec

- Annealing: 60 °C for 40 sec

- Extension: 72 °C for 30 sec

**Figure 1:**
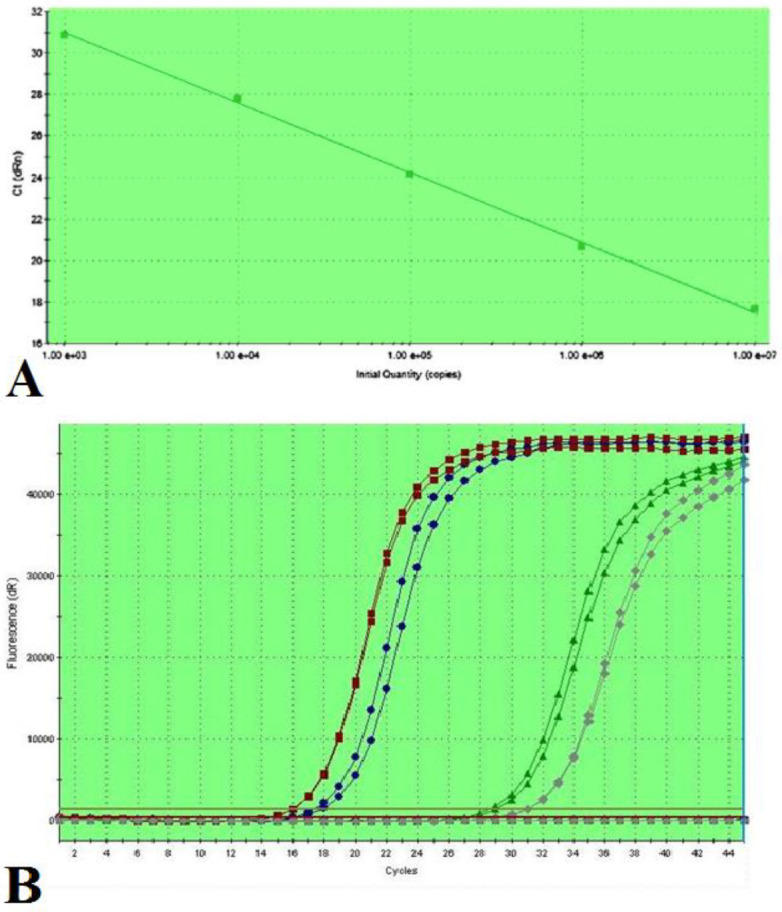
A: The standard curve shows 98% efficacy for DPT amplification. B: Amplification curves related to 2 tumoral tissues and their corresponding controls

Then a melt curve analysis was performed, in which the temperature was increased from 55 °C to 95 °C, at a linear rate of 0.2 °C/sec. Fluorescent measurement was achieved at each cycle at the end of the extension. The real-time PCR efficacies were 98% and 95% for DPT and GAPDH amplifications, respectively. The standard curve and amplification curves of some samples are shown in Figure 1. 

Dermatopontin (Gene of interest (GOI)) expression was calculated by employing the ^ΔΔ^CT method.

$\mathrm{Normalized sample}/\mathrm{control}=\frac{{\left(1+E_{\mathrm{GOI}}\right)}^{{-∆\mathrm{CT}}_{\mathrm{GOI}}}}{{\left(1+E_{\mathrm{GAPDH}}\right)}^{{-∆\mathrm{CT}}_{\mathrm{GAPDH}}}}$

Normalization of the resulting cycle threshold (Cq) values of the target mRNAs was done using the Cq values of the housekeeping gene (GAPDH) as an internal control, in the same samples.

When the calculated DPT mRNA expression was ≤2 folds in tumor tissue compared with corresponding tumor-free tissue, it was considered to be under-expressed. Overexpression was regarded as ≥2 fold increase in DPT mRNA expression of tumoral tissue in comparison with the non-tumoral tissue. The range in-between was interpreted as no change in expression.

***Mutation analysis of DPT gene***

*DNA extraction*

In the case of DPT underexpression (based on real-time PCR results), tumoral DNA was extracted using the proteinase K method. In brief, 100 µl of digestion buffer (containing 50 mM Tris [pH=8.5], 1000 M EDTA, 1% laureth 12 or 0.5% Tween 20) plus 10 µl of proteinase K were added to each 10 mg of tumoral tissue. After 3 hr incubation at 55 °C, followed by 10 min at 95 ºC, the mixture was centrifuged (at 13400 rpm) and the upper fraction, containing DNA, was removed. The quality and quantity of the extracted DNAs were assessed by an ultraviolet spectrophotometer and agarose gel electrophoresis. 

*PCR*

Four sets of oligonucleotide primers for exons of dermatopontin were designed (Table 1). Primer-BLAST was performed, confirming the specificity of the primers (www.blast.ncbi.nlm.nih.gov). Polymerase chain reactions were performed using Applied Biosystems 2720 thermal cycler (Applied Biosystems Inc., USA). The reaction mix in each tube included 1 µl of dNTP (4×10 mM) (Genet Bio, Korea), 1 µl of Taq DNA polymerase (5 U) (Genet Bio, Korea), 5 µl of PCR 10X buffer (Genet Bio, Korea), 2 µl of MgCl_2_ (25 mM) (Genet Bio, Korea), 1 µl of each primer (10 pmol/µl), 2 µl of DNA, and 37 µl of deionized H_2_O in a total volume of 50 µl.

The thermal cycle conditions of the reactions were as follows:

Initial denaturation: 5 min at 95 °C

35 cycles:

- Denaturation: 30 sec at 95 °C

- Annealing: 45 sec at the specific annealing temperature of each set of primers based on Table 2

- Extension: 45 sec at 72 °C

Final Extension: 5 min at 72 °C

Using agarose gel electrophoresis, the quality and quantity of the products were assessed.

*Sequencing*

Amplified PCR fragments were sequenced by the Sanger method employing the Applied Biosystems 3730XL capillary electrophoresis machine (Applied Biosystems Inc., USA). Data were analyzed using the Seqscape software package (Applied Biosystems Inc., USA). 

***Statistical analysis***

A Chi-square test with a 95% confidence interval was performed for comparing the qualitative variables. Exact chi-square test and Fisher’s exact test were used as an alternative when indicated by the analysis. Since the quantitative variables such as age and fold changes in DPT expression had a normal distribution, we used Student’s t-test after having examined the equality of variances with Levene’s test and one-way ANOVA to compare these variables in different groups. For evaluating the correlation between age and fold changes in DPT expression, Pearson’s correlation coefficient was employed. A value of *P*<0.05 was considered statistically significant. The statistical analyses were performed using the IBM SPSS 20.0 statistical package (SPSS, Inc, Chicago, IL, USA).

## Results

***Cancer profiling array data***

Matched Tumor/Normal expression Array 71.4% (5/7) of colon cancer samples showed down-regulation and 28.6 % (2/7) showed up-regulation of DPT in comparison with their normal counterparts. Cancer Profiling Array I 85.3% (29/34) of tumoral colon samples showed down-regulation, 8.8% (3/34) revealed up-regulation and 5.9% (2/34) showed no change in DPT expression, comparing their matched normal colonic tissue. In this array, 4 of the samples were matched with the metastatic tumor tissues. 75% (3/4) of the metastases showed down-regulation and 25% (1/4) showed no change in DPT expression. 50% (2/4) of these metastatic samples showed more than 2-fold down-regulation in DPT expression in contrast to their primary site tumors.

***Real time PCR data***

Twenty-four out of 38 patients with colorectal adenocarcinoma were male (M/F ratio: 1.71). The median age was 53.5 years ranging from 21 to 86, mean: 53.6. 41% of patients were under 50 years old. Most of the tumors were located in distal parts of the large intestine (74% (n=28)), had moderate degrees of differentiation (59% (n=22)), and were in stage II (59% (n=22)).

Of 38 studied cases, 14 (36.8%) showed underexpression of DPT mRNA in their tumoral tissues compared with corresponding tumor-free tissues, while 12 (31.6%) cases had no change in expression of DPT, and the other 12 (31.6%) tumors cases overexpressed the DPT gene. Figure 1 shows the mean and standard deviation of fold changes in DPT expression of these groups (*P*<0.001).

The age of patients in different DPT expression groups including overexpression, underexpression, and no change in expression, were not significantly different (*P*=0.45), and there was no correlation between age and fold changes in DPT expression (r= -0.16, *P*=0.36). The DPT expression by fold changes and expression groups corresponding to the descriptive characteristics of patients and different subgroups of tumors are summarized in Tables 2 and 3. There were no significant differences in DPT expression between different groups. 45.5% of advanced tumors (stages III & IV) overexpressed DPT, while only 26.9% of low-stage tumors (I & II) showed DPT overexpression (*P*=0.44). Furthermore, DPT overexpression was seen in 47.7% of tumors having lymph node involvement, while 28% of N0 tumors had such expression patterns (*P*=0.47).

Regarding the tumor location, DPT underexpression was more common in proximal colon tumors than distal ones (50% vs. 32%), although the differences were not statistically significant.

The results of sequencing of exonic regions of the DPT gene in 12 cases with DPT under-expression are shown in Table 4. In one case there was a point mutation at exon 2 (Val 139 Ser), which could be the cause of DPT under expression in this case. A frameshift mutation at exon 4 (215 ins A), was found in another case that can explain the DPT under expression. Some other changes were found in several cases, but they may not influence gene expression level according to their high frequency, or not affect the amino acid sequence or their location in far distance from the coding region (Table 4).

**Table 1:** Primer sequences used in real-time PCR and PCRs of DPT exons

**Primer**		**Sequence 5′→3′**	**Amplicon size (b.p.)**
Primer sequences used in real-time PCR:			
**DPT**	***F1***	ATATTCCTGCTGGCTAAC	168
	***R1***	ACAGTCGTATTCAGTCATC	
**GAPDH**	***F1***	GGAAGGTGAAGGTCGGAGTCA	101
	***R1***	GTCATTGATGGCAACAATATCCACT	
Primer sequences used in PCRs of DPT exons:			
**E1**	***F1***	TCAAAAACATCTGGAAGCC	485
	***R1***	ATTCCTTGAGAGTCTAGCAG	
**E2**	***F2***	GGTCTGTGTATGTGTCACTGTTC	248
	***R2***	AAGCTAGAGCACTTCCTAGG	
**E3**	***F3***	GTCTTCTGTTTGCTCCCAGC	323
	***R3***	GAAATTTTCACTTGTATTCTACG	
**E4**	***F4***	TGT TTC CCC AGCAGC GATTC	425
	***R4***	AGCATGCGCACTGTATATG	

DPT: Dermatopontin

DPT: Dermatopontin

**Table 2:** Comparison of DPT expression (by fold changes) between different groups of patients (real-time PCR data)

		**DPT expression ** **(** *fold changes* **)**	
	N*	**Mean ± SD** ^†^	***P-value***
**Gender**			
*Male*	24	-0.59 ± 3.63	*0.6*
*Female*	14	0.24 ± 5.16	
**Age**			
*Under 50 years*	14	0.44 ± 3.48	*0.5*
*50 years & older*	20	-0.52 ± 4.76	
**Grade**			
*Well-differentiated*	14	-0.02 ± 4.48	*0.85*
*Moderately differentiated*	22	-0.52 ± 4.23	
*Poorly differentiated*	1	1.66 ± 4.22	
**Stage**			
*I & II*	26	-0.77 ± 4.59	*0.2*
*III & IV*	11	0.90 ± 3.05	
**Lymph node involvement**			
*N0*	25	-0.54 ± 4.53	*0.56*
*N>0*	12	0.28 ± 3.62	
**Tumor location**			
*Proximal*	10	-1.66 ± 4.95	*0.39*
*Distal*	28	0.08 ± 4.26	

*N: number of samples; †Mean±SD: mean±standard deviation

DPT: Dermatopontin

**Table 3:** Comparison of DPT expression (based on DPT expression category) between different groups of patients (real-time PCR data)

	**DPT expression change**			
	*Under expression*	*No change*	*Overexpression*	***P-value***
**Gender**				
*Male*	37.5% (9)*	37.5% (9)*	25.0% (6)*	*0.49*
*Female*	35.7% (5)*	21.4% (3)*	42.9% (6)*	
**Grade**				
*Well differentiated*	28.6% (4)*	28.6% (4)*	42.9 (6)*	*0.61*
*Moderately differentiated*	40.9% (9)*	31.8% (7)*	27.3% (6)*	
*Poorly differentiated*	0% (0)*	100% (1)*	0% (0)*	
**Stage**				
*I & II*	38.5% (10)*	34.6% (9)*	26.9% (7)*	*0.66*
*III & IV*	27.3% (3)*	27.3% (3)*	45.5% (5)*	
**Lymph node involvement**				
*N0*	36% (9)*	36% (9)*	28% (7)*	*0.75*
*N>0*	33.3% (4)*	25% (3)*	4.7% (5)*	
**Tumor location**				
*Proximal*	50% (5)*	30% (3)*	20% (2)*	*0.59*
*Distal*	32% (9)*	32% (9)*	36% (10)*	

*Number of samples

DPT: Dermatopontin

**Table 4:** Results of exon sequencing of DPT gene in tumoral tissues of 12 colorectal cancer cases with DPT under expression

**Case number**	**Exon 1**	**Exon 2**	**Exon 3**	**Exon 4**
1	Thr 80 Thr homo*	-	-	215 del G het†
2	-	-	-	215 ins A het†
3	-	-	+41 IVS^‡^ 3 A>G	-
4	-	-	-	215 del G het†
5	Thr 80 Thr homo*	-	-	215 del G het†
6	-	Val 139 Ser	-	215 del G het†
7 to 12	-	-	-	-

* homo: homozygote; † het: heterozygote; ‡ IVS: intronic region

DPT: Dermatopontin

**Figure 2:**
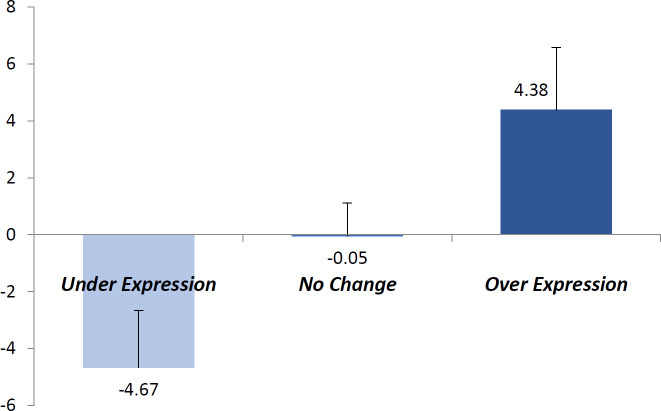
The mean and standard deviation of fold changes in DPT expression of different groups of patients based on DPT expression category (real-time PCR data)

**Figure 3:**
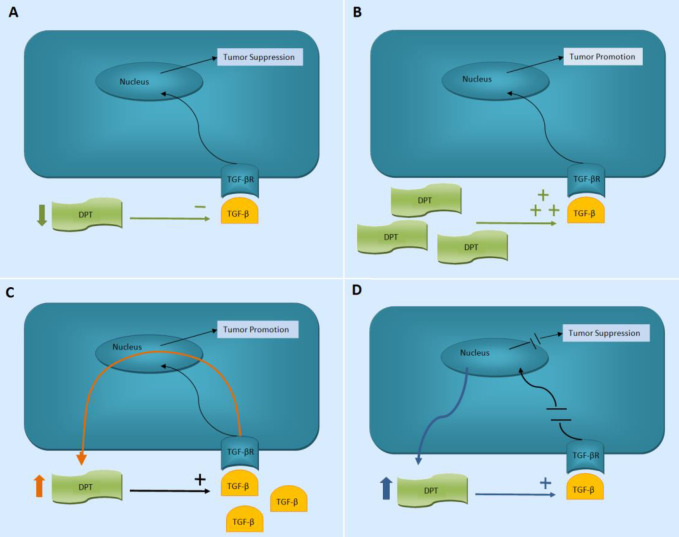
A: Probable way of DPT tumor suppression. B, C, and D: Theoretical possibilities for DPT overexpression in colorectal cancer. A primary increase in DPT may induce tumor aggressiveness through the TGF-β signaling pathway (B). High levels of TGF-β may lead to DPT overexpression (C). Diminished activity of the TGF-β pathway in the cell may induce DPT overexpression to restore the pathway (D)

## Discussion

DPT is a small molecule of the extracellular matrix functioning in modification of collagen fibrillogenesis, cell adhesion, increasing the biological activity of TGF-β, and induction of cell quiescence (7, 9, 11, 13). In the present study, the expression level of DPT in colon adenocarcinoma was analyzed using CPA arrays and real-time PCR, and aggregately in 60.8% (48/79) of the examined tumors the expression level was reduced. Since DPT can induce quiescence and increase TGF-β activity, which in turn acts as a tumor suppressor in many cancers, this finding may be in line with the introduction of a tumor suppressor role for DPT (Figures 3-A). TGF-β pathway undergoes changes in lots of colon cancers mainly through receptor mutation (TGF-βRII) and mutations in components of the intracellular signaling pathway (Smad4) (12, 20). Decreased DPT and consequently reduction in the biological activity of TGF-β may be another moderating factor in the TGF-β pathway in those tumors in which, receptor and post-receptor mutations, have not been found. However, to clarify this issue, studies are required that simultaneously examine the changes in the TGF-β pathway and DPT expressions. In line with our findings, decreased expression of DPT has been demonstrated in oral squamous cell carcinoma, hepatocellular carcinoma and related cell lines, breast and ovarian cancers, as well as uterine leiomyomas (14-19). In a study on hepatocellular carcinoma and related cell lines, decreased expression of DPT mRNA and protein, despite increase in TGF-β protein (using IHC) was observed (15). Also, in another study on uterine leiomyoma, reduced DPT and increased TGF-β expression were observed compared with normal myometrium (17). Although the observed increase in TGF-β can result in neutralization of the effect of reduced DPT on the function of this pathway, it should be noted that DPT could play a role in tumorigenesis through other pathways including quiescence induction and affecting cellular adhesion. In addition, unlike earlier observations that suggested an inhibitory role for TGF-β in carcinogenesis, it is now believed that TGF-β inhibits early tumorigenesis as it may cause progression of cancer in later tumor stages (20, 21). Thus, the above findings may be due to increase in TGF-β in late steps, without any relationship with the anti-TGF-β effect of reduced DPT, and only indicate the accumulation of proper characteristics for further tumor growth during tumor progression steps.

Another finding of our study was the increase in DPT expression in about 21.5% (17/79, CPA and real-time PCR data aggregately) of the tumors. As already mentioned, TGF-β has been considered as both tumor suppressor and promoter, and this is evident during the development of colon cancer. Colon carcinoma cells isolated from metastatic sites proliferate in response to TGF-β, while growth of cells derived from moderate to well-differentiated tumors in the primary site is inhibited by TGF-β (22, 23). In addition, strong staining of colonic tumor cells at the primary site for TGF-β has been associated with increased risk of recurrence (23). It is debated when and how this shift in the function of TGF-β from inhibitor to promoter occurs (20). The mutation in Smad4 (a molecule involved in the intracellular signaling pathway of TGF-β) has been proposed as a hypothesis in this regard; the absence of which has caused aggressive tumor behavior in response to TGF-β. Accordingly, our data can be interpreted in several ways. First, similar to an increase in TGF-β, increased DPT can play an effective role in the induction of tumor aggressiveness through the TGF-β signaling pathway; leading to selection of cancer cells with this feature during cancer development (Figures 3-B). Since TGF-β induces increased expression of DPT, the overexpression of DPT in some of our tumoral samples may be an effect of increased TGF-β on this protein (Figure 3-C). Another theory is the cell effort to restore the loss of or diminished TGF-β activity in cells having mutations in TGF-βR or Smad, through increased expression of DPT (Figures 3-D). Deeper studies in this field, especially the evaluation of the mechanisms of alteration in DPT expression will help to clarify the issue.

In the present study, Using CPA, two of the metastatic site tumors showed down regulation, comparing to their corresponding primary site tumors. Furthermore, there was no significant difference in the expression level of DPT in different grades and stages of cancer, using real-time PCR. In 28% of tumors with no lymph node involvement, DPT was overexpressed compared with 42% of tumors having metastatic lymph nodes. Also, while 45% of higher-stage cancers showed increased expression of DPT, only 27% of lower-stage tumors (Stages I and II) demonstrated DPT overexpression. The lack of significant differences between these groups may be due to the limited sample size. To our knowledge, few studies have dealt with the role of DPT in tumor behavior. In the study of Yamatoji *et al*. on oral squamous cell carcinoma using IHC, the regional lymph node metastasis was significantly higher in tumors with no immunoreactivity for DPT (DPT negative), compared with tumors that showed positive DPT labeling (14). Researchers assumed the role of DPT in cell adhesion in this regard (14). Another study using quantitative real-time PCR showed that DPT was down-regulated in hepatocellular carcinoma and that the low expression level of DPT was correlated with more metastasis and worse survival. They found that DPT suppressed metastasis through α3β1 integrin-Rho GTPase signaling (24). Whether DPT reduces aggressive behavior through its role in cell adhesion or increases the aggressiveness of cancer by increasing the activity of TGF-β and enhancement of its tumor promoter effect, demands more research in this field. One possibility is that the role of DPT is affected by the changes in other components of the TGF-β pathway, and different functions of it are executed in different settings of cooperating molecules.

In this study, reduced expression of DPT was seen in 50% of proximal colon tumors, while only 33% of distal colon tumors showed a reduction in DPT expression. This finding complies with the pattern of changes in the TGF-β signaling pathway in colon cancer. According to reports, mutations in TGF-βRII are more common in tumors of the proximal colon than the distal colon (20). This has raised the possibility that TGF-βRII may have no role in rectosigmoid carcinogenesis, or disturbance in another component in the TGF-β signaling pathway may be involved in tumorigenesis of these regions (20).

To find the underlying cause of change in DPT gene expression, its exons were sequenced in fresh tumoral cases that showed DPT under expression. A point mutation, as well as a frameshift mutation, was found in 2 cases which may explain the under expression of DPT in these cases. Mutations that could effectively reduce DPT expression were not found in other cases. So, mutation does not seem to be a major cause of DPT under expression.

Yamatoji *et al*. and Fu *et al*. found epigenetic factors as the reason for DPT under expression in oral squamous cell carcinoma and hepatocellular carcinoma, respectively (14, 24). There is a need for future research to evaluate the role of epigenetic factors in changes in DPT expression in CRC.

## Conclusion

In this study, decreased expression of DPT was observed in 60.8% of colon cancers, and increased expression of DPT was seen in 21.5% of tumors. Given our current knowledge, the tumor suppressor effect of DPT is mainly performed by increasing the activity of TGF-β, these findings suggest that DPT may act as another component of the TGF-β signaling pathway, in the pre-receptor step through increased biological activity of TGF-β, and may account for changes in this pathway in the remaining colon cancers in which changes in TGF-β pathway have not been demonstrated. Studies with larger sample sizes that simultaneously survey the changes in various components of the TGF-β pathway, as well as DPT, would help elucidate the role of these molecules in colon carcinogenesis.
